# Quantitation of microRNA-92a in colorectal adenocarcinoma and its precancerous lesions: Co-utilization of *in situ* hybridization and spectral imaging

**DOI:** 10.3892/ol.2014.2813

**Published:** 2014-12-18

**Authors:** I WENG LAO, FENGYUN CUI, HONGGUANG ZHU

**Affiliations:** 1 Department of Pathology, School of Basic Medical Sciences, Fudan University, Shanghai 200032, P.R. China; 2 Division of Surgical Pathology, Huashan Hospital, Fudan University, Shanghai 200032, P.R. China; 3 Institutes of Biomedical Sciences, Fudan University, Shanghai 200032, P.R. China

**Keywords:** *in situ* hybridization, multispectral imaging microscopy, colorectal adenocarcinoma, microRNA, miR-92a, quantitation

## Abstract

The expression level of microRNA (miR)-92a has been proven to increase during the development of colorectal adenocarcinoma (CA) and has been verified at the cellular, plasma and fecal levels by various quantitative methods. However, a method to quantitate the expression level using tissue sections has not been established. To do this, *in situ* hybridization (ISH) and multispectral imaging microscopy (MSI) were introduced to quantitate miR-92a expression on the microscopic level. ISH of miR-92a was first performed on 34 tissue samples of CA and adenomas with high-grade and low-grade intraepithelial neoplasms, while 31 paralesional normal tissue samples were defined as the control. Subsequently, a MSI technique was applied to quantitate the hybridization signal in terms of optical density (OD) at the visible wavelength. A t-test with unequal variance was used to examine the statistical significance between the groups. Despite all 34 tissue sections demonstrating at least partial positivity of miR-92a expression following ISH, visual grading was inconclusive. As such, the signal of ISH was transformed in terms of OD and further analyzed by employing the MSI system. A statistically significant difference was observed between the expression levels of miR-92a in CA and the paralesional normal controls. By contrast, a poor correlation was revealed between visual and spectral grading. The co-utilization of ISH and MSI generated a legible observation in the expression level of miR-92a, revealing the dynamic change in miR-92a expression in the progression of the disease and providing important information for further functional investigation.

## Introduction

microRNA (miRNA/miR) is a ~22 nt long, non-coding small RNA that is essential in post-transcriptional regulation and has been suggested to directly and indirectly regulate genesis, differentiation, proliferation, growth and apoptosis in eukaryotes, particularly in carcinogenesis (1–4).

As one well-established technique used to quantitatively study micromolecules, *in situ* hybridization (ISH) is favored due to its ability to directly observe the spatial expression of the studied candidate. Through utilizing radioactive isotopes, fluorophores or chromophores as indicators to label any single complementary DNA or RNA strand so that the strand serves as a probe and targets specific counterstrands of interest, ISH allows the visualization of minute changes within cells (5,6). Variant forms of ISH have been derived and widely applied in the field of diagnostics, as well as in fundamental studies (6).

Multispectral imaging microscopy (MSI) is an advanced method applied in the analysis of macro- and microscopic samples from a three-dimensional aspect (7,8). MSI is a quantitative technique that adds spatial resolution to the spectral images when analyzing samples, first performed by assigning intensity as a function of wavelength, then by acquiring an image as a constellation of pixel units, with each pixel unit classified by its spectral signature, and genuinely creating an image cube that contains spectral and spatial information (8,9). This does not only resolve the expression of numerous components within a single cell, but also generates information on any dynamic changes from normal to aberrant cells, providing a simple yet convenient method for biomedical studies (9–11).

The present study attempted to observe the changes in the expression level of miR-92a at a tissue level, as miR-92a had been previously proven to increase at the cellular, plasma and fecal levels during the development of colorectal adenocarcinoma (CA) (12–14). It was found that quantitation of miRNAs was not realized by ISH independently, as miR-92a was only expressed in cells with a differentiation in level, which was consistent with the findings of Liang *et al* (15). Therefore, a secondary approach of employing a spectral imaging technique followed by ISH was developed in an attempt to quantitate the changes in level of miR-92a expression.

## Materials and methods

### Tissue samples

In total, 34 tissue samples of colorectal lesions collected from the surgically resected specimens obtained from colorectal cancer patients who underwent hemicolorectomies and colorectomies at Huashan Hospital (Shanghai, China) between 2009 and 2012, that represented three consecutive stages were grouped as follows: 10 samples of low-grade intraepithelial neoplasia (LGIN), 11 samples of high-grade intraepithelial neoplasia (HGIN) and 13 samples of CA. For the internal normal control, 31 normal paralesional tissue samples were obtained, and were divided into three groups: Nine normal controls for LGIN (LGIN-N), 10 for HGIN (HGIN-N) and 12 for CA (CA-N). Three samples possessed no adjacent normal mucosa samples. Diagnoses were confirmed by two senior pathologists in the Department of Pathology (School of Basic Medical Sciences, Fudan University, Shanghai, China). The study was approved by the ethics committee of Shanghai Medical College, Fudan University. Written informed consent was obtained from all patients.

### miRNA in situ hybridization

All samples were fixed with 10% buffered formalin and were paraffin embedded. Each sample block was sectioned into 6-μm thick slices and mounted on charged slides. Hybridization was performed following the procedures reported previously (16,17). Briefly, the slides were deparaffinized and dehydrated with xylene and an ascending gradient of ethanol and the slides were then rehydrated using phosphate-buffered saline (PBS) at pH 7.4. Subsequently, proteinase digestion was performed using proteinase K (15 μg/ml; Exiqon, Vedbaek, Denmark) for 8 min at 37°C, and the slides were washed with 3× PBS at pH 7.4. Hybridization was performed with a locked nucleic acid (LNA)-modified, 5′-digoxigenin-labeled probe of miR-92a (sense, 5′-ACAGGCCGGGACAAGTGCAATA-3′; Exiqon) at a concentration of 40 nM, using PTC-100™ Programmable Thermal Controller (MJ Research, Inc., Waltham, MA, USA) at 55°C for 1 h. Following hybridization, a stringency wash was performed on the slides with a descending gradient of saline-sodium citrate (5×, 2× and 0.2×) at 4°C (17). Blocking was performed using anti-digoxigenin alkaline-phosphatase combined with sheep serum (DIG Nucleic Acid Detection kit; Roche Diagnostics, Indianapolis, IN, USA) at room temperature for 1 h. The slides were then stained with nitro blue tetrazolium/5-bromo-4-chloro-3-indolyl phosphate (NBT/BCIP; SIGMAFAST™ BCIP^®^/NBT; Sigma-Aldrich, St. Louis, MO, USA) for signal development, and the nuclei were counterstained with methyl green.

### Visual grading of ISH result

The average expression of miR-92a on the slides was visually and individually graded by two pathologists using a light microscope at ×200 magnification (Carl Zeiss Microscopy GmbH, Göttingen, Germany). A four-tier scoring system was devised according to the cytoplasmic staining intensity: i) Negative, unstained cytoplasm or cytoplasm exhibiting only background color; ii) weak positive, cytoplasm exhibiting a light indigo color; iii) moderate, cytoplasm exhibiting a weak to moderate indigo stain; and iv) strong positive, cytoplasm exhibiting a dark indigo stain.

### Image acquisition and signal interpretation by MSI system

**Figure 1:**
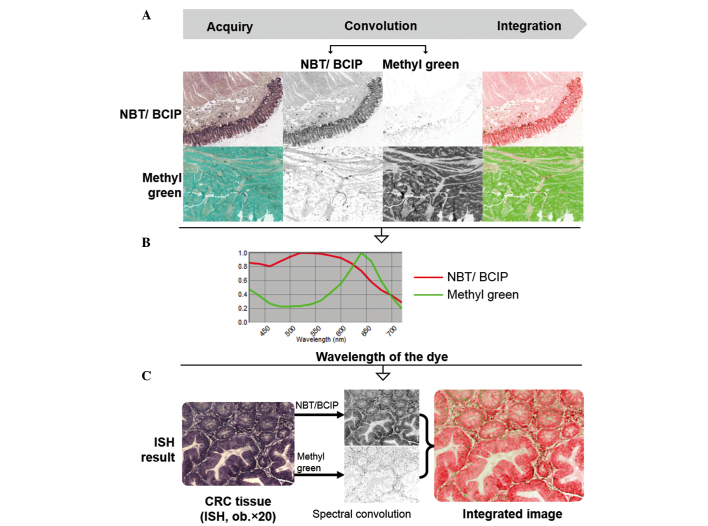
Principle of multispectral imaging technology in analyzing the expression level of miRNA. (A and B) Applying the CRi-Nuance™ Multispectral Imaging System. Dyes with a wavelength between 420 and 720 nm (NBT/BCIP and methyl green) were acquired as references (microphotographs at ×50 magnification) and (C) microphotographs of tissues that underwent ISH, including colorectal adenocarcinoma (CA) tissues, were spectrally resolved to detect the expression level of miR-92a (magnification, ×200). NBT/BCIP, nitro blue tetrazolium/5-bromo-4-chloro-3-indolyl phosphate; ISH, *in situ* hybridization; miR/miRNA, microRNA.

**Figure 2:**
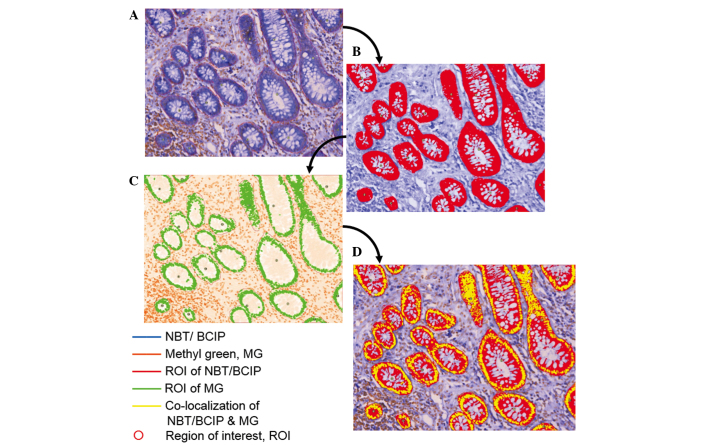
CRi-Nuance™ Multispectral Imaging System was applied to analyze the expression level of miR-92a in colorectal adenocarcinoma and its precancerous lesions. Applying the function of co-localization of Nuance to analyze the expression level of miR-92a in colorectal tissue, the expression pattern and intensity were then generated as raw data for further statistical assay. (A) A different color was appointed to represent NBT/BCIP and MG, and the region of interest was circled. In the image, blue represents NBT/ BCIP, orange represents MG and red circles represent the region of interest. (B) The threshold of ROI was adjusted and the orange color represents the area where miR-92a is expressed. (C) The threshold of MG was adjusted and the green color represents the area stained with the dye. (D) The threshold of the channel was adjusted, indicating co-localization of NBT/BCIP and MG. The yellow color represents data generated based on the pixel area, followed by calculation of the concentration of NBT/BCIP in the cytoplasm with the formula as provided, which represented the expression of miR-92a. NBT/BCIP, nitro blue tetrazolium/5-bromo-4-chloro-3-indolyl phosphate; ROI, region of interest; MG, methyl green.

Using the CRi-Nuance™ Multispectral Imaging System (Cambridge Research and Instrumentation Inc., Woburn, MA, USA), one representative unstained slide from each of the three investigated groups was selected and stained with NBT/BCIP and methyl green, respectively. These slides were used to obtain spectral references, as shown in Fig. 1. For each tissue sample, a region of interest (ROI) demonstrating the average visual grading was randomly selected within the site of miR-92a expression, and images were then acquired under ×200 magnification using a charge-coupled device (CCD) camera to accompany the imaging system. The average NBT/BCIP optical density (OD) signal was detected from each field, and the average signal and pixel areas were further generated within the ROI by the Nuance analyzer (Cambridge Research and Instrumentation Inc.) as shown in Fig. 2. Signal interpretation was also devised into a four-tiered system by a quartile cut-off value according to the minimum (0.20825) and maximum (0.9455) OD: i) Negative, OD ≤0.377; ii) weak positive, OD 0.378–0.5105; iii) moderate, OD 0.5106–0.68025; and iv) strong positive, OD >0.68025. The average signal was calculated from the area using unit pixels and the concentration of miR-92a in the cytoplasm, as represented by the NBT/BCIP concentration, as follows: Average signal = Total signal / Area pixels. Provided that miR-92a concentration per cell = total NBT / BCIP concentration per cell, miR-92a concentration per cell = miR-92a concentration within (cytoplasm + nucleus): NBT/BCIP cytoplasm = [total signal (full image) − total signal (overlap)] / [total signal (full image) − total signal (overlap)]. Thus, miR-92a concentration within the cytoplasm = total NBT/BCIP concentration per cell − NBT/BCIP concentration within the nucleus, which was therefore, the concentration of NBT/BCIP in the cytoplasm, the non-overlapped area with NBT/BCIP expression per cell.

### Statistical analysis

Using Microsoft Excel 2007 (Microsoft, Redmond, WA, USA), a t*-*test with unequal variance was used to compare LGIN, HGIN, CA and their paralesional normal counterparts, LGIN-N, HGIN-N and CA-N, to estimate the differential expression of miR-92a. Statistical significance was defined as the two-tailed P-value for rejecting the hypothesis of zero correlation and indicated using P<0.05. A scatter diagram was plotted by Graphpad Prism^®^ (Version 5.0; GraphPad Software, Inc., La Jolla, CA, USA) to illustrate the association between the visual grading of ISH and the OD value obtained from ISH-MSI.

## Results

### miR-92a expression level is visually indeterminable by ISH in CA, LGIN, HGIN and each of their corresponding paralesional normal controls

**Figure 3:**
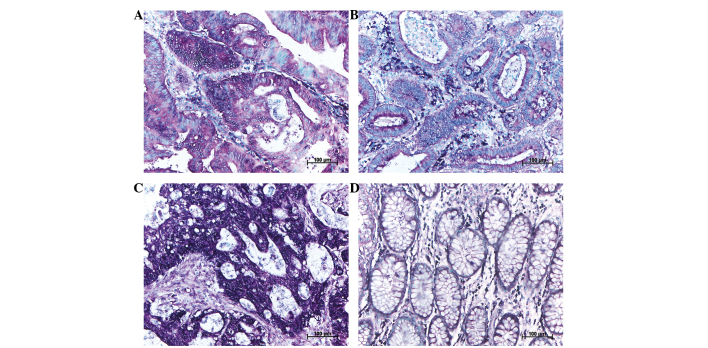
Expression of miR-92a in colorectal adenocarcinoma (CA) and its precancerous lesions, revealed by *in situ* hybridization (magnification, ×200). (A) High-grade intraepithelial neoplasia. (B) Low-grade intraepithelial neoplasia. (C) CA. (D) Normal control of CA. Positive expression of miR-92a is represented by nitro blue tetrazolium/5-bromo-4-chloro-3-indolyl phosphate, with the nuclei counterstained by methylgreen. Scale bar, 100 μm.

All tissue sections indicated at least partial positivity for miR-92a expression. Therefore, no section was graded as negative in Fig. 3. The median visual grading (VD*x*) of staining intensity was moderate for CA, CA-N, LGIN and LGIN-N, while for HGIN and HGIN-N the VD*x* was moderate to positive and moderate, respectively.

### miR-92a expression level significantly differs between CA, LGIN, HGIN and their paralesional normal controls, as determined by MSI analysis

**Figure 4:**
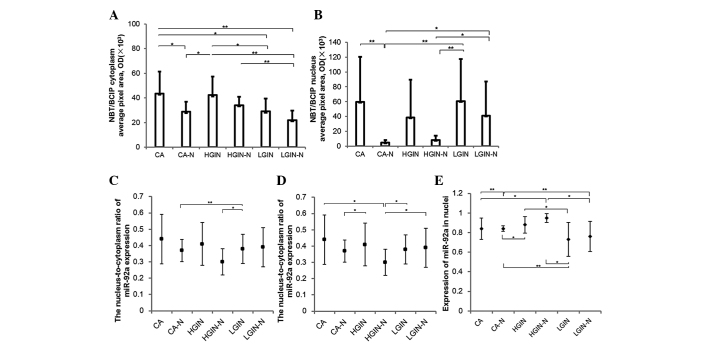
Multispectral imaging analysis of the expression level of miR-92a in 34 samples of colorectal adenocarcinoma (CA) and its precancerous lesions (CA, n=13; CA-N, n=12; HGIN, n=11; HGIN-N, n=10; LGIN, n=10; and LGIN-N, n=9). (A) The average expression level of miR-92a in the cytoplasm of the tissue, determined by pixel area. (B) The average expression level of miR-92a in the nuclei of the tissue, determined by pixel area. (C) The average expression intensity of miR-92a in the cytoplasm of the tissue, determined by OD. (D) The nucleus-to-cytoplasm ratio of miR-92a expression in each group. (E) The expression of miR-92a in the nuclei of each group. The above data is calculated by the formula mentioned in the Materials and methods section. Each sample was repeated three times and the error bar represents the standard deviation. ^*^P<0.05; ^**^P<0.01. CA, colorectal carcinoma; CA-N, CA paralesional normal tissue; HGIN, high-grade intraepithelial neoplasia; HGIN-N, HGIN paralesional normal tissue; LGIN, low-grade intraepithelial neoplasia; LGIN-N, LGIN paralesional normal tissue; NBT/ BCIP, nitro blue tetrazolium/5-bromo-4-chloro-3-indolyl phosphate; OD, optical density.

Using the MSI system, the expression level of miR-92a in the cytoplasm was isolated and further analyzed following the aforementioned calculations. Statistically significant differences were observed in the expression level of miR-92a between various categories of CA, CA-N, LGIN and HGIN, and their paralesional normal controls, LGIN-N and HGIN-N, respectively. In particular, for the average pixel area of the cytoplasm covered with NBT/BCIP, which determined the expression area of miR-92a, a significant difference was revealed when CA was compared with CA-N (P=0.020) and LGIN (P=0.018), when HGIN was compared with CA-N (P=0.027) and LGIN (P=0.018), when LGIN-N was compared with HGIN (P=0.012) and CA (P=0.0014), and when HGIN-N was compared with LGIN-N (P=0.0009). In the nuclei, the average pixel area of NBT/BCIP presented a significant difference in miR-92a expression when LGIN-N was compared with CA-N (P=0.019) and HGIN-N (P=0.031), when CA was compared with CA-N (P=0.0099), when CA-N was compared with LGIN (P=0.004) and when HGIN-N was compared with CA (P=0.014) and LGIN (P=0.006). For the expression intensity of miR-92a, denoted by NBT/BCIP, a significant difference was revealed when LGIN was compared with HGIN-N (P=0.012) and when CA-N was compared with LGIN (P=0.0006). For the nuclei to cytoplasm ratio of miR-92a expression intensity, a significant difference was revealed when HGIN-N was compared with CA (P=0.017), LGIN (P=0.038) and LGIN-N (P=0.046). Also, there was a significant difference when CA-N was compared with HGIN (P=0.028). For the expression area of miR-92a over the ROI, a significant difference was revealed when HGIN-N was compared with CA (P=0.032), LGIN (P=0.002) and LGIN-N (P=0.011), when HGIN was compared with CA-N (P=0.032) and LGIN (P=0.011), when CA was compared with CA-N (P=0.005), and when CA-N was compared with LGIN (P=0.001) and LGIN-N (P=0.003). All these data are summarized and extrapolated in Fig. 4.

### ISH combined with MSI legibly evaluates changes of miR-92a expression level in CA, LGIN, HGIN and their paralesional normal controls

**Figure 5:**
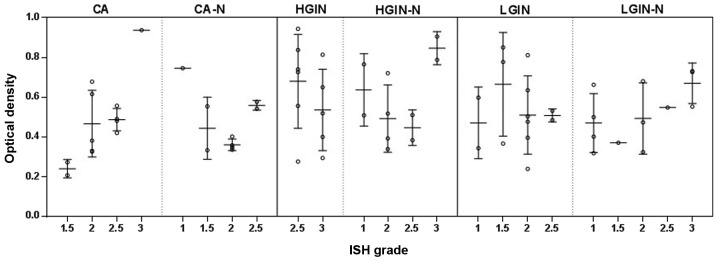
Comparison of stain intensity between spectral imaging (optical density) and visual grading (ISH grade) for all samples. All values were plotted and are shown as the mean ± standard deviation (horizontal line). No intra- or inter-group colinearity was observed. CA, colorectal carcinoma; CA-N, CA paralesional normal tissue; HGIN, high-grade intraepithelial neoplasia; HGIN-N, HGIN paralesional normal tissue; LGIN, low-grade intraepithelial neoplasia; LGIN-N, LGIN paralesional normal tissue; ISH, *in situ* hybridization.

The visual grading of the ISH results from the 34 samples was compared with the OD obtained by the combination of ISH and MSI in order to determine whether this modified technique could better evaluate the change in miR-92a expression. A scatter diagram plotted the visual grading of ISH (x-axis) against the OD value obtained from ISH-MSI (y-axis), as shown in Fig. 5. Linear regression analysis revealed no significant correlation between the two quantification methods (rs=0.25; P>0.05).

## Discussion

The latest development of miRNA quantitation techniques initially focused on the cellular level to the body fluids, and included quantitative PCR assays (18,19), next-generation sequencing (21), MSI (20) and miRNA sensing in living cells based on peptide nucleic acid and nano-graphene oxide (21). Until recently, no concrete method was designed to directly observe changes in the expression of miRNAs through immediate tissue observation. In several studies, the miR-92a expression level has been revealed to be elevated during the progression of CA (22–24). The hybridization signal on tissue sections did not provide a similar conclusion, as it revealed that miR-92a is universally, but unevenly, expressed in all studied samples, in CA, LGIN, HGIN and their paralesional normal controls. Nevertheless, this finding was consistent with the results of the study by Liang *et al*, which noted the expression of miR-92a in normal and cancerous organs (15). Compared with other common tumor indicators that are expressed in an all or nothing manner in cells, the expression characteristics of miR-92a resulted in an inconclusive, moderate, visual scoring for all studied samples containing colorectal lesions at various stages, with the exception of between HGIN and its normal control, inferring that an additional approach is required to evaluate the changes in the expression level of miR-29a.

Since the visual judgment of staining intensity was based on color development, MSI was recruited in the present study. MSI encompassed the ability to discriminate a wide color spectrum and evaluate the concentration of dye, as shown when this method was analogously applied in a study quantitating thymidylate synthase in CA (11). CISH was performed prior to imaging analysis, followed by the detection of expression level by the MSI system, using chromogens within the spectrum of 420 to 700 nm at the visible wavelength. The expression details, area and intensity, were calculated to precisely and objectively evaluate the differential expression of miR-92a from normal to cancerous colonic tissues. The results from quantitating the expression of miR-92a in CA, LGIN, HGIN and their paralesional normal controls with ISH-MSI revealed a significant difference between certain considered criteria, particularly the pattern and concentration of miR-92a expression in the nuclei and cytoplasm, assessed by the average pixel area and intensity from OD, respectively. The accretion of miR-92a from the precancerous lesions to CA by the nucleus-to-cytoplasm ratio and its amplitude of expression over the area studied demonstrated consistency with the findings of studies at the cellular, plasma and fecal levels (22–24), demonstrating that the observation of changes in the expression level of miRNA may be assessed at the microscopic level.

In the present study, objective visualization of the changes in miRNA expression was achieved with the implementation of CISH and MSI. ISH of miRNAs uses factitiously designed LNA-oligonucleotide probes that steadily and specifically detect minute concentrations of miRNA (25,26). Likewise, hybridization using chromogens possesses several advantages compared with its fluorescence counterpart, as chromogen-labeling permits tangibility of specific cell types, which enables selection and observation of the region of interest for study under the light microscope, including the recognition of cancerous and normal colonic cells in the present study, since fluorophore-labeling techniques ambiguously discriminate between various cell types in the dark field. In addition, CISH requires a longer preservation period, with the generation of well-defined signals and the ability to select the definite regions of interest that radioactive and fluorescence counterparts cannot guarantee (27,28). However, MSI demonstrates the capability of measuring multiple analytes carrying specific spectra at one time (29), which accurately resolves and relatively quantitates the changes in the miRNA expression level of various cell types, which conventional ISH would not achieve. This provides an objective and easy to use platform that is widely applied in a plethora of fields in biomedical research, including cytology, immunohistochemistry and nanoparticle studies (7,8,30,31). Out of the available spectral imaging technology, the system employed in the present study used liquid crystal tunable filters, which confer advantages of a narrow spectral bandwidth, 7–20 nm, with an improved spectral resolution and a changeable wavelength at different ranges, such as the visible wavelength at 420–720 nm and near infra-red at 850–1,800 nm, which allows evaluation for the fluorescence- and chromogen-based samples (9,29). Coupling with a cooled scientific-grade monochrome CCD camera, the system produces excellent signal discrimination and image quality (9,11).

Regardless of the frequent suggestions that the reproducibility of MSI could be undermined by artifacts generated during specimen preparation and the standardization of any parameters taken into the analysis, it has been suggested that, under strict and controlled conditions, artificial biases could be reduced to an optimal extent (32).

In conclusion, the co-utilization of ISH and spectral imaging analysis could validate the expression of miRNA through spectral and spatial evaluation, providing an improved understanding of its functions during the progression of diseases, generating valuable information for further study, in order to supply an effective and efficient diagnostic parameter for future clinical practice.
